# Patient-centric analysis of *Orientia tsutsugamushi* spatial diversity patterns across Hainan Island, China

**DOI:** 10.1371/journal.pntd.0012909

**Published:** 2025-03-18

**Authors:** Chuanning Tang, Yi Huang, Gaoyu Wang, Liying Xue, Xiaoyuan Hu, Ruoyan Peng, Jiang Du, Jinyan Yang, Yi Niu, Wanxin Deng, Yibo Jia, Yijia Guo, Siqi Chen, Nan Ge, Liyuan Zhang, Fahui Wang, Yongguo Du, Yueping Wang, Long Sun, Jasper Fuk-Woo Chan, Kwok-Yung Yuen, Biao Wu, Feifei Yin

**Affiliations:** 1 Hainan Medical University-The University of Hong Kong Joint Laboratory of Tropical Infectious Diseases, Key Laboratory of Tropical Translational Medicine of Ministry of Education, Academician Workstation of Hainan Province, School of Basic Medicine and Life Sciences, Hainan Medical University, Haikou, Hainan, China; 2 NHC Key Laboratory of Systems Biology of Pathogens, National Institute of Pathogen Biology, Chinese Academy of Medical Sciences & Peking Union Medical College, Beijing, China; 3 Department of Infectious Diseases, The Second Affiliated Hospital of Hainan Medical University, Haikou, Hainan, China; 4 Department of Infectious Diseases, The First Affiliated Hospital of Hainan Medical University, Haikou, Hainan, China; 5 State Key Laboratory of Emerging Infectious Diseases, Department of Microbiology, and Carol Yu Centre for Infection, Li Ka Shing Faculty of Medicine, The University of Hong Kong, Pokfulam, Hong Kong Special Administrative Region, China; 6 The University of Hong Kong-Shenzhen Hospital, Shenzhen, Guangdong, China; 7 Hainan General Hospital, Hainan Affiliated Hospital of Hainan Medical University, Haikou, Hainan, China; 8 Hainan Public Health Clinical Center, Haikou, Hainan, China; 9 Department of Clinical Laboratory, Center for laboratory Medicine, Hainan Women and Children’s Medical Center, Hainan Medical University, Haikou, Hainan, China; University of Virginia School of Medicine, UNITED STATES OF AMERICA

## Abstract

**Background:**

Scrub typhus, traditionally caused by *Orientia tsutsugamushi*, is a re-emerging public health concern within the Tsutsugamushi Triangle. Despite growing awareness, prevention strategies remain inadequate on Hainan Island, China, where scrub typhus poses a significant threat, especially in field-related environments.

**Methodology/principal findings:**

Gene flow analysis of the *tsa56* gene and multilocus sequence typing (MLST) were conducted on 156 previously confirmed scrub typhus cases from 2018 to 2021 across Hainan Island. By integrating published datasets, we identified 12 major sub-genotypes and traced their origins, revealing that these sub-genotypes share origins with isolates from Southeast Asia and coastal provinces and island of China, but also demonstrate unique local adaptations across all isolates. Alpha diversity index analysis was applied across administrative regions to identify hotspot regions. This analysis showed that nine out of the detected fourteen administrative regions, particularly along the northern and western coastlines and inland areas, exhibited relatively high genetic diversity, with the highest incidence observed in Qiongzhong, a centrally located city. Related major sequence types were mapped, and distances between locations were estimated, showing that identical MLST sequence types were observed to transfer across distances of 23 to 125 km between different sites on the island. Pathogen density was analyzed using quantitative real-time PCR targeting the *tsa56* gene. Without accounting for potential confounding factors or dataset limitations, the Karp_B_2 sub-genotype showed a significant increasing trend in pathogen density with prolonged fever duration, while Gilliam sub-genotypes exhibited a slower or even declining trend.

**Conclusions/significance:**

These findings emphasize the urgent need for targeted public health interventions, particularly focusing on vulnerable populations in rural and agricultural areas of nine key administrative regions where high genetic diversity and pathogen spread were observed. Additionally, this study provides valuable insights into the transmission dynamics and infection progression of scrub typhus, using gene flow analysis and multilocus sequence typing to identify major sub-genotypes.

## Author summary

Scrub typhus, caused by *Orientia tsutsugamushi* transmitted via larval mite bites, is a re-emerging public health threat on Hainan Island within the Tsutsugamushi Triangle, offering a unique setting for studying disease transmission and genetic diversity. Utilizing gene flow analysis of the *tsa56* gene and multilocus sequence typing, we identified 12 major sub-genotypes and traced their origins by integrating new and published data. Our analysis revealed that Hainan’s sub-genotypes share origins with the second and third recombinant generations of the *tsa56* gene from isolates in Southeast Asia and China’s coastal regions, while also exhibiting unique local adaptations. Notably, the highest incidence and genetic diversity were observed in the inland city of Qiongzhong, and nine key regions showed significant genetic diversity with evidence of long-distance transmission of identical MLST sequence types, prompting us to consider whether birds play a role in the transmission process. The newly classified Karp_B_2 sub-genotype exhibited a more pronounced increasing trend in pathogen density during fever, while Gilliam sub-genotypes showed less distinct trends. These findings regarding pathogen density highlight general patterns rather than definitive conclusions, given the potential influence of confounding variables and the limited sample size. These findings underscore the urgent need for targeted public health interventions in rural and agricultural areas of Hainan island where high genetic diversity and pathogen spread are prevalent. The broadly applicable genotyping methods used provide a foundation for more effective disease management and control strategies, focusing on hotspot regions and specific genotypes.

## Introduction

Scrub typhus, an acute febrile infectious disease caused by the obligate intracellular bacterium *Orientia tsutsugamushi* (*O. tsutsugamushi*), is transmitted to humans through bites from larval mites or chiggers, and presents a re-emerging public health threat on Hainan Island, China, as well as in the broader Asia-Oceania region [1–3]. Traditionally endemic to the ‘Scrub Typhus Triangle’, which includes South Asia, Southeast Asia, East Asia, and parts of Oceania — such as northern Australia and certain Pacific Islands [4,5] — scrub typhus has also been reported outside the Triangle in a relatively broader geographical range, including Chile, the Arabian Peninsula, and parts of Africa. Cases reported outside the Triangle have been attributed to Orientia species, such as *O. chuto*, which have not been documented within the traditional Triangle [6–9]. Studies have shown that approximately one in five people across Asia are seropositive for *O. tsutsugamushi*, with seroprevalence rates ranging from 9.3% to 27.9%, and a median of 22.2%, underscoring a significant public health risk in this region [10]. Despite this risk, scrub typhus remains insufficiently researched, particularly in terms of its diagnosis and prevention.

*O. tsutsugamushi* is currently classified into three main antigenic groups—namely Karp, Kato, and Gilliam—based on the serotypes of the prototype strains [11,12]. Several potential protective antigens have been identified as vaccine targets, including a 56 kDa membrane protein (TSA56) encoded by the *tsa56* gene, a 47 kDa transmembrane protein, a surface cell antigen ScaA, a 22 kDa protein, and a 110 kDa protein from Karp [13–16]. Among these biomarkers, TSA56 protein, which is highly variable and constitutes about 20% of the bacterial proteome, plays a crucial role in *O. tsutsugamushi* adhesion by interacting with fibronectin [17–19]. Variability of the *tsa56* gene sequence is closely linked to the antigenic diversity of *O. tsutsugamushi* genotypes, primarily defined by four variable domains (i.e., VD I–IV) within the TSA56 protein. These domains, spanning 16–40 amino acids in hydrophilic regions, are critical for differentiating *O. tsutsugamushi* genotypes through the analysis of the 56 kDa protein [20–22]. Additionally, to develop a more cost-effective detection method suitable for use under Biosafety Level 2 laboratory conditions required for *O. tsutsugamushi* [23], a nested PCR targeting a 418–453 nucleotide segment of the *tsa56* gene — covering Variable Domain II (VD II) and VD III — has been widely utilized without the need to amplify the entire gene [24–27]. This nested PCR has proven accurate for genotyping strains such as Karp, Boryong, Gilliam, TA763, Kawasaki, and certain Kato strains. However, its accuracy for genotyping Shimokoshi and some other Kato strains remains unclear [28].

While the *tsa56* gene plays a crucial role in genotyping, genomic analysis and Multi-Locus Sequence Typing (MLST) provide a broader perspective on genetic diversity, advancing our understanding of the evolution and epidemiology of *O. tsutsugamushi* [29–32]. Significant genetic differences exist among *O. tsutsugamushi* strains, including variations in genome size, GC content, genome structure, and the distribution of functional genes [33,34]. Pan-genome analyses indicate that the *O. tsutsugamushi* genome is ‘closed,’ with genetic diversity primarily arising from gene mutations, duplications, and divergences rather than from the acquisition of exogenous genes through processes such as transduction, transformation, and conjugation [35]. This pattern of intra-species lateral gene transfer, which is relatively uncommon among other bacteria, may contribute to the evolutionary adaptation of *O. tsutsugamushi* to its hosts and environments and is closely related to its pathogenicity [35,36]. In particular, the diversification of the *tsa56* gene is driven by frequent point mutations followed by multiple recombination events, observed across four generations of 17 proto-genotype sequences [37]. However, these recombination events in *tsa56* do not necessarily determine the virulence of specific genotypes [38].

Previously, we have investigated the bacterial load associated with the TA763, Karp, and Gilliam groups on Hainan Island, identifying untyped Gilliam strains [39]; however, that study did not sufficiently address the genotype classification. To overcome this limitation, the present study provides a patient-centric analysis of the spatial diversity patterns and pathogen density of *O. tsutsugamushi* across Hainan Island, integrating *tsa56* genotypes with MLST to enhance our understanding and management of the disease. The findings are expected to have relevance beyond the local population of Hainan Island, as the genotyping methods employed in this study are broadly applicable.

## Methods

### Ethics statement

The Ethics Committee of Hainan Medical University granted approval for this study under reference number [HYLL-2020-061]. Written informed consent was obtained from all participants, either directly from adult patients or from a parent or legal guardian for those under 18 years of age.

### Sample collection

This study was conducted on patients with suspected *Orientia tsutsugamushi* infection who presented with fever of unknown origin at four hospitals on Hainan Island from June 2018 to May 2021. The hospitals involved included the First Affiliated Hospital of Hainan Medical University, the Second Affiliated Hospital of Hainan Medical University, Haikou People’s Hospital in the northern part of the island, and Qiongzhong People’s Hospital in the central mountain district.

Patients were enrolled based on the following inclusion criteria: axillary temperature ≥37.5°C, accompanied by at least one of the following symptoms—eschar, skin rash, lymphadenopathy, hepatomegaly, and/or splenomegaly—or a history of field exposure within three weeks prior to symptom onset. Whole blood and serum samples were collected immediately upon admission. These samples were transported to the laboratory within 48 hours using cold chain logistics and stored at -80°C until testing.

The sample set in this study was re-analyzed from the dataset previously reported in our earlier publication, covering the period from July 2018 to November 2021 [39]. This overlap ensures a comprehensive analysis while maintaining the integrity of the data.

### Diagnostic testing

Two diagnostic methods were employed to confirm *O. tsutsugamushi* infection: a Rapid Serological Diagnostic Test (Colloidal Gold, WANTAI Inc., China) and a PCR assay targeting the hypervariable region of the *tsa56* gene. The Rapid Serological Diagnostic Test was conducted on blood samples to detect antibodies against *O. tsutsugamushi*. Additionally, DNA was extracted from blood samples for PCR amplification of a 483 bp fragment of the *tsa56* gene to identify the presence of the pathogen, as previously described [24]. A sample was considered positive for *O. tsutsugamushi* if either both IgG and IgM antibodies were positive or the PCR result was positive. The combined use of both diagnostic methods aimed to maximize detection accuracy.

### Multilocus sequence typing

Genetic profiling of *O. tsutsugamushi* was performed on 97 out of 115 PCR-positive samples. Nested PCR was conducted to amplify seven canonical conserved genes: *gpsA*, *mdh*, *nrdF*, *nuoF*, *ppdK*, *sucB* and *sucD*. The primers and PCR conditions used were reported in previous published studies [29–31]. Sanger sequencing was used to analyze the PCR products, and sequence types (STs) were assigned based on the allele profiles submitted to the PubMLST database (https://pubmlst.org/organisms/orientia-tsutsugamushi/) [40]. Clonal complexes (CCs) were identified based on the MLST data, defined as groups of STs that evolved from a central ST through single-locus variants (SLVs). Additionally, expanding clone groups (CGs) were identified, each comprising multiple isolates sharing the same ST. The relationships between STs were visualized using the phyloviz software (version 2.0).

### Typing of the *tsa56* gene and phylogenetic classification

Genomic DNA was extracted from whole blood samples using the QIAamp DNA Mini Kit (QIAGEN, Hilden, Germany) following the manufacturer’s instructions. Nested PCR was performed using primers designed to amplify a 483 bp fragment of the *tsa56* gene, as previously described [24,28]. The first round of the nested PCR was carried out in a 50μl reaction mixture containing 1μl of KOD One PCR Master Mix (TOYOBO CO., LTD, Japan), 0.2μM of each forward and reverse primer (tsu-34 and tsu-55), and 1μl of DNA template. The thermal cycling conditions involved an initial denaturation at 98°C for 5 minutes, then 30 cycles of denaturation at 98°C for 10 seconds, annealing at 55°C for 5 seconds, and extension at 68°C for 5 seconds, concluding with a final extension at 68°C for 1 minute. The second round of nested PCR was performed using 1μl of the first-round PCR product with primers tsu-10 and tsu-11 under the same conditions, except with an annealing temperature of 62.5°C. For positive samples, the PCR products were purified using the Omega Gel Extraction Kit (Omega Bio-tek, USA) and subjected to Sanger sequencing. The sequences of the *tsa56* genes obtained by Sanger sequencing were analyzed using BioEdit software version 7.2.5 (Ibis Biosciences, Carlsbad, CA, USA) and compared with sequences from two reference databases [28,37] using the BLAST algorithm.

Phylogenetic analysis of *O. tsutsugamushi*, based on partial sequences of the *tsa56* gene and the concatenated sequences of seven conserved genes used in MLST, was conducted separately using the Neighbor-Joining (NJ) method in MEGA X software, with 1000 bootstrap replications (Pennsylvania State University, PA, USA).

### Spatial analysis of genetic diversity

The spatial distribution of *O. tsutsugamushi* genotypes identified across Hainan Island was analyzed to map each genotype to specific geographic locations. Geographic mapping was performed manually, where genotypes were plotted and color-coded on a physical map based on the sampling locations. The Shannon Diversity Index was calculated for different geographic regions across Hainan Island in R using the ‘vegan’ package. Paired comparisons of diversity indices between these regions were conducted using Student’s t-test.

### Pathogen density analysis

Pathogen density was quantified using quantitative real-time PCR (qPCR) targeting the *tsa56* gene, a major antigenic determinant widely used for the diagnosis and quantification of *O. tsutsugamushi* infection [39]. Pathogen density was calculated as the copy number of the *tsa56* gene per milliliter of blood, based on plasmid standard curves.

To assess the relationship between infection duration and pathogen density, linear regression models were utilized, with the log-transformed pathogen density as the dependent variable and infection duration (in days) as the independent variable. For each genotype, a linear model was fitted using the ‘lm’ function in R, and the results were summarized by examining *P*-value.

Scrub typhus severity was classified into mild (no organ dysfunction), moderate (one organ dysfunction), and severe (two or more organ dysfunctions) categories, following previously established criteria [41,42]. Organ dysfunction criteria included pulmonary (bilateral infiltrates with hypoxia or mechanical ventilation requirement), cardiovascular (systolic blood pressure < 80 mmHg, severe anemia, or cardiac issues), central nervous system (seizures, stroke, meningitis, or coma), hepatic (total bilirubin ≥ 42.7μmol/L), renal (creatinine ≥ 177μmol/L), and digestive (gastrointestinal hemorrhage). Genotypes with sufficient information were visually represented accordingly.

### Statistical analysis and visualization

Statistical analyses were conducted using RStudio (version 2023.06.0). Visualizations, including tanglegrams and box plots, were generated using R packages such as ggplot2 and phytools.

## Results

### Population genetic structure of *O. tsutsugamushi* in clinical cases across Hainan Island

This study used 156 confirmed scrub typhus cases (29.4%) out of 531 suspected cases, as included in previously published data from four hospitals in Hainan over a three-year period [39]. Detailed test results by hospital are summarized in S1 Table. Based on population data from 2018 to 2020 [43], the annualized incidence rates of scrub typhus varied across Hainan Island, with the highest in Qiongzhong (0.79 per 10,000 per year) and most regions reporting rates below 0.1 per 10,000. Haikou, despite having the second highest number of infections, had a low rate of 0.03 per 10,000 due to its large population. No data or cases were available for regions such as Sanya, Sansha, Wuzhishan, and Lingshui (S2 Table). Annual infection rates were mapped to the specific residential locations of the cases (S2 Fig). Among the *O. tsutsugamushi* cases, 76 (48.7%) were farmers, 16 (10.3%) did fieldwork, 24 (15.4%) lived in rural areas, and 24 (15.4%) in urban/suburban areas, including 3 travelers from mainland China. Living conditions were unknown for 16 (10.2%) patients.

**Fig 1:**
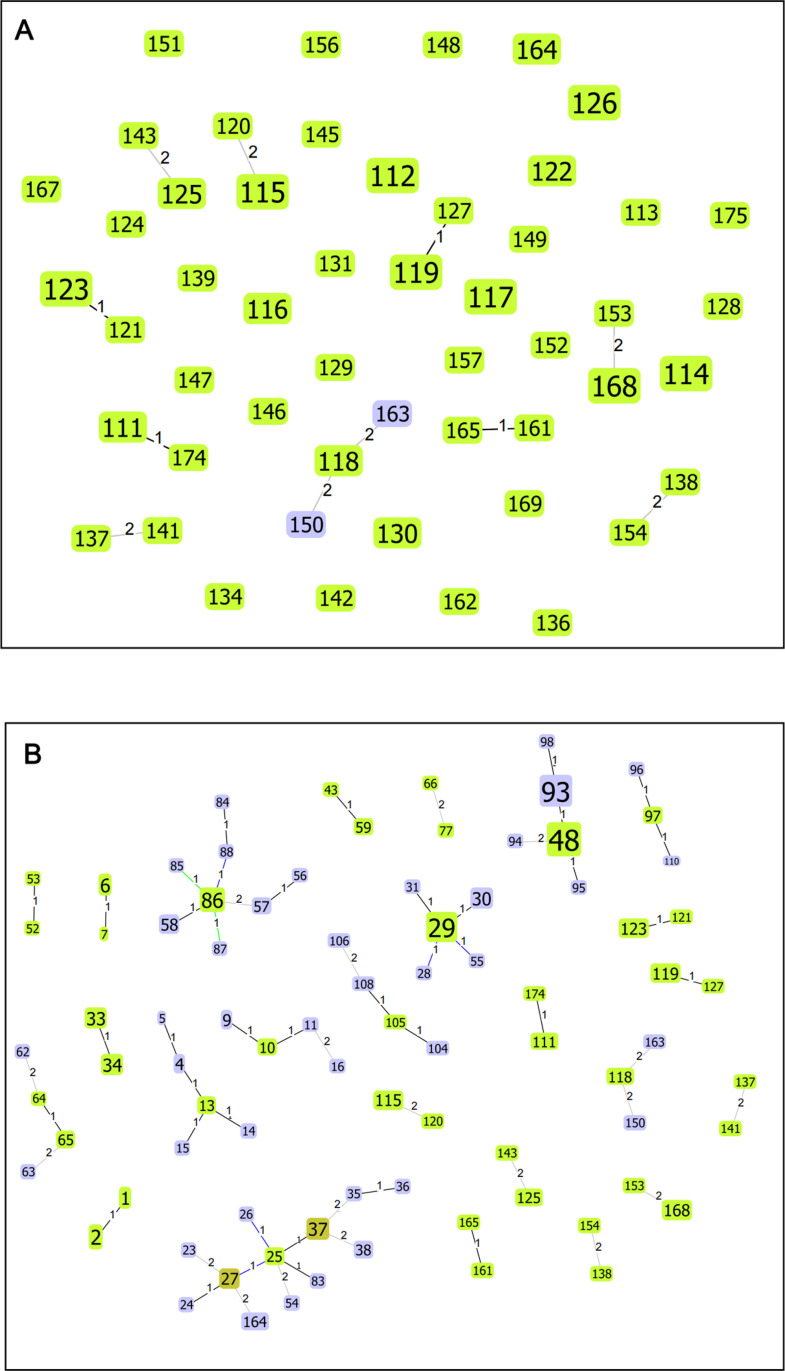
Population genetic structure of *O*. *tsutsugamushi* in Hainan Island and comparison with other regions using MLST analysis. (A) *O. tsutsugamushi* population structure on Hainan Island (sample size n = 74) as revealed by geoBURST analysis, identifying newly reported sequence types (STs) and their associated clonal complexes (CCs). (B) Comparison of *O. tsutsugamushi* populations from Hainan Island (sample size n = 74) with those from other regions in the pubMLST database (n = 235), focusing only on clonal complexes (CCs). Regional breakdown: Thailand (n= 83, ST1-ST44); Papua New Guinea (n = 1, ST45); Burma (n = 1, ST46); Australia (n = 1, ST47); Japan (n = 2, ST20, ST49); Laos (n = 74, ST50-ST92, ST1, ST4, ST9, ST25, ST29, ST30, ST37); South Korea (n = 63, ST48, ST93-ST98); Malaysia (n = 2, ST99, ST100); Bangladesh (n = 8, ST101-ST108); Hainan Island, China (n=74, ST111-ST175).

Genetic profiling of seven canonical conserved genes was successfully performed on 97 out of 115 *O. tsutsugamushi*-positive samples identified via PCR testing of the *tsa56* gene. In addition, mixed infections, indicated by double peaks at multiple loci (n≥2) in nested-PCR Sanger sequencing results of the conserved genes, were detected in 23 patients (23.7%). The 74 samples without evidence of mixed infections were used for subsequent population genetic structure analysis, with MLST analysis of canonical conserved genes performed. A total of 51 STs, ranging from ST111 to ST175, were identified. These STs are newly reported and have not been previously documented in the PubMLST database, as detailed in Table 1. Four clonal complexes, comprising sequence types that evolved from a central ST through single-locus variants, were identified: CC111, CC119, CC123, and CC161/165. These SLV complexes include ST pairs with a distance label of 1, covering ST111–ST174, ST119–ST127, ST121–ST123, and ST161–ST165. Fig 1A illustrates both SLV and double-locus variant (DLV) relationships, with five pairs of STs and a group of three STs identified as DLVs. Additionally, 12 expanding clone groups were observed, each consisting of 2 or 3 isolates sharing the same ST.

**Table 1:** Sequence Types (STs) and Genotypes of 74 *O. tsutsugamushi* Isolates from Hainan Island. Isolate names prefixed with “HMU-” represent samples stored in Hainan Medical University. CCs refer to clonal complexes, defined as groups where any ST matches the central genotype at six loci. CG indicates a clone group.

ID	ST	MLST loci							CCs or CG	Genotype
		*gpsA*	*mdh*	*nrdB*	*nuoF*	*ppdk*	*sucB*	*sucD*		
HMU-5	111	49	34	40	47	50	39	45	CC111	Karp_B_1
HMU-44	111	49	34	40	47	50	39	45	CC111	Karp_B_1
HMU-6	112	34	35	7	48	47	35	48	CG112	Karp_A_2
HMU-17	112	34	35	7	48	47	35	48	CG112	Karp_A_2
HMU-34	112	34	35	7	48	47	35	48	CG112	Karp_A_2
HMU-7	113	28	9	7	49	47	2	50		JG_C_1
HMU-9	114	28	9	41	1	47	4	47	CG114	JG_C_1
HMU-29	114	28	9	41	1	47	4	47	CG114	JG_C_1
HMU-74	114	28	9	41	1	47	4	47	CG114	JG_C_1
HMU-10	115	27	34	7	50	50	12	52	CG115	Karp_C
HMU-100	115	27	34	7	50	50	12	52	CG115	Karp_C
HMU-106	115	27	34	7	50	50	12	52	CG115	Karp_C
HMU-11	116	50	1	41	49	51	4	55	CG116	JG_C_1
HMU-69	116	50	1	41	49	51	4	55	CG116	JG_C_1
HMU-13	117	51	9	42	51	45	41	49	CG117	TA763_A
HMU-41	117	51	9	42	51	45	41	49	CG117	TA763_A
HMU-97	117	51	9	42	51	45	41	49	CG117	TA763_A
HMU-15	118	52	34	42	35	17	2	45	CG118	Karp_B_2
HMU-95	118	52	34	42	35	17	2	45	CG118	Karp_B_2
HMU-43	119	49	9	14	55	46	43	47	CC119	Karp_A_1
HMU-75	119	49	9	14	55	46	43	47	CC119	Karp_A_1
HMU-103	119	49	9	14	55	46	43	47	CC119	Karp_A_1
HMU-19	120	55	34	7	49	50	12	52		Karp_C
HMU-20	121	13	4	43	48	47	6	45	CC123	Karp_A_2
HMU-26	122	53	36	14	52	45	6	50	CG122	Karp_Qiong
HMU-73	122	53	36	14	52	45	6	50	CG122	Karp_Qiong
HMU-27	123	34	4	43	48	47	6	45	CC123	Karp_A_2
HMU-49	123	34	4	43	48	47	6	45	CC123	Karp_A_2
HMU-101	123	34	4	43	48	47	6	45	CC123	Karp_A_2
HMU-66	124	49	34	40	49	46	43	45		Karp_B_1
HMU-30	125	54	37	44	53	48	38	5	CG125	JG_B
HMU-76	125	54	37	44	53	48	38	5	CG125	JG_B
HMU-31	126	26	9	42	54	49	40	50	CG126	Karp_A_2
HMU-38	126	26	9	42	54	49	40	50	CG126	JG_Qiong
HMU-94	126	26	9	42	54	49	40	50	CG126	JG_Qiong
HMU-32	127	49	9	14	55	48	43	47	CC119	Karp_A_1
HMU-33	128	53	38	14	52	44	42	53		Karp_A_2
HMU-59	129	52	41	42	35	50	4	45		Karp_B_2
HMU-36	130	55	34	45	55	50	2	47	CG130	Karp_C
HMU-55	130	55	34	45	55	50	2	47	CG130	Karp_C
HMU-37	131	28	39	41	56	47	2	45		Karp_C
HMU-42	134	28	9	20	1	27	6	54		JG_C_1
HMU-45	136	26	34	7	35	47	43	51		Karp_A_2
HMU-46	137	54	40	7	48	47	43	44		JG_B
HMU-47	138	50	1	46	57	48	4	55		JG_C_1
HMU-48	139	34	4	41	49	51	4	45		Karp_A_2
HMU-50	141	54	40	43	48	47	6	44		JG_B
HMU-51	142	52	34	8	57	17	4	45		Karp_B_2
HMU-52	143	54	37	42	53	48	2	5		JG_B
HMU-56	145	53	36	45	58	50	2	50		Karp_Qiong
HMU-57	146	28	9	14	49	45	6	50		JG_C_1
HMU-60	147	53	41	42	52	51	2	50		Karp_Qiong
HMU-61	148	34	4	14	48	45	6	46		Karp_A_2
HMU-62	149	51	9	7	51	47	43	49		TA763_A
HMU-63	150	52	9	42	35	17	41	45		Karp_B_2
HMU-64	151	34	35	42	48	45	2	48		Karp_A_2
HMU-65	152	49	9	7	55	47	35	47		Karp_A_1
HMU-67	153	54	40	14	57	48	37	44		JG_B
HMU-68	154	50	1	8	47	48	4	55		JG_C_1
HMU-70	156	53	9	41	49	51	4	42		Karp_A_2
HMU-71	157	50	1	14	52	44	6	55		Karp_B_2
HMU-77	161	10	8	40	59	17	2	14	CC161/165	JG_C_2
HMU-78	162	55	42	40	60	32	2	47		Karp_C
HMU-79	163	20	43	42	35	17	2	45		Karp_B_2
HMU-80	164	10	8	8	59	10	6	42	CG164	JG_C_2
HMU-82	164	10	8	8	59	10	6	42	CG164	JG_C_2
HMU-81	165	10	8	40	59	17	36	14	CC161/165	JG_C_2
HMU-83	167	10	9	8	59	10	2	50		JG_C_2
HMU-85	168	54	40	8	57	48	4	44	CG168	JG_B
HMU-90	168	54	40	8	57	48	4	44	CG168	JG_B
HMU-102	168	54	40	8	57	48	4	44	CG168	JG_B
HMU-87	169	10	34	3	59	10	2	5		JG_C_2
HMU-98	174	49	34	40	47	50	37	45	CC111	Karp_B_1
HMU-99	175	1	4	47	61	14	2	39		Kato_A

**Fig 2:**
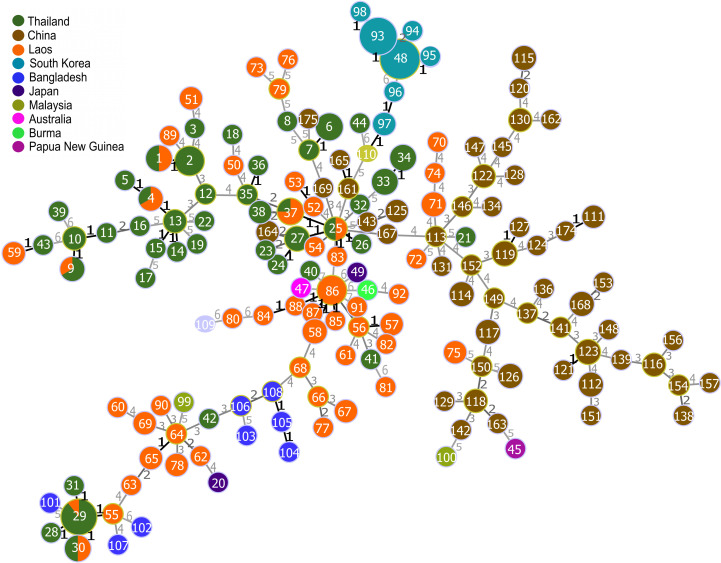
Clonal complexes of *O. tsutsugamushi* population from Hainan Island, China, and other regions using phyloviz with full minimum spanning tree (MST). The numbers in the figure represent unique clone groups based on sequence types (STs) defined in the MLST database. ST node colors represent different regions: Hainan Island (sample size n = 74), Australia (n = 1), Bangladesh (n = 8), Burma (n = 1), Japan (n = 2), Laos (n = 74), Malaysia (n = 2), Papua New Guinea (n = 1), South Korea (n = 63), and Thailand (n = 83).

Visualization of sequence data for all 51 STs from 74 Hainan isolates, along with 110 STs from 235 Asian-Oceania isolates available in PubMLST, was performed using Phyloviz with geoBURST for DLV analysis and a Full Minimum Spanning Tree, as shown in Figs 1B and 2. The Asian-Oceania isolates included samples from Australia (1 isolate), Bangladesh (8 isolates), Burma (1 isolate), Japan (2 isolates), Laos (74 isolates), Malaysia (2 isolates), Papua New Guinea (1 isolate), South Korea (63 isolates), and Thailand (83 isolates). *Orientia tsutsugamushi* isolates from Hainan Island displayed unique genetic markers and formed distinct clonal complexes, with no direct genetic connections to isolates from other regions in Asia-Oceania at a distance label of 1 (equivalent to SLV). However, at a distance label of 2, ST164 was linked to ST27, with all three ST27 isolates originating from in Thailand. Phyloviz analysis indicated that ST25 is likely a shared, closely related recent type among strains from Thailand, Laos, and Hainan, China (Fig 2). This analysis also uncovered several possible lineage connections within Hainan Island when the criteria for linking STs were relaxed. Most isolates from Hainan Island formed a large independent cluster, distinct from the lineages observed in Thailand, Laos, Bangladesh, and South Korea. However, sequence types ST125, ST143, ST167, ST169, ST161, ST165, and ST164 from Hainan Island were found to be connected to ST25 and ST27 from Thailand, while ST175 was linked to ST7 from Thailand. Further analysis revealed that these isolates belong to the Gilliam and Kato_A lineages, respectively.

### Gene flow analysis of *tsa56* genotypes reveals lineage origins of *O. tsutsugamushi* on Hainan Island

To further investigate the genetic relationships and origins of these lineages, *tsa56* gene typing was conducted, and the recombination generations were classified based on the hypervariable regions described in two key literature sources [28,37]. Additional details on the classification of generations are provided in S1 Fig. The sequences analyzed ranged in length from 420 to 453 nucleotides and were compared with 533 sequences from previous studies. Within the five primary evolutionary groups — Karp, Gilliam, Kato, TA763, and Shimokoshi — Shimokoshi was absent from Hainan Island. Of the 17 unique genotypes identified previously, seven were detected on Hainan Island, all exhibiting at least 99% nucleotide identity. These include second-generation genotypes such as Karp_C (found in Japan, South Korea, Taiwan of China, Thailand, and Papua New Guinea), Karp_B (Vietnam, Taiwan of China, Thailand), and Karp_A (Cambodia, Zhejiang of China, Fujian of China, Taiwan of China, Thailand, Vietnam, Malaysia). Third-generation genotypes like JG_C (found in Taiwan of China and Zhejiang of China, Cambodia, Vietnam, Thailand), JG_B (Taiwan of China, Thailand) from Gilliam, TA763_A (Taiwan of China, Vietnam, Thailand) from TA763, and Kato_A (Cambodia, Taiwan of China) from Kato were also identified. Additionally, Karp_Qiong and JG_Qiong were identified, showing 97.76% nucleotide identity to Karp_C (HQ660203, South Korea) and 97.86% to JG_B (GQ332754, Taiwan of China), respectively.

First-generation genotypes such as Saitama (from Karp group, found in Japan, Taiwan of China, and South Korea), Boryong (from Karp, identified in South Korea, Japan, Guangdong of China, and Taiwan of China), Kawasaki (from Gilliam, identified in South Korea, Japan, Taiwan of China), TD (from Gilliam, identified in Taiwan of China), Shimokoshi (from Shimokoshi, identified in Japan), and TA686 (from Shimokoshi, identified in Thailand) were absent. Second-generation genotypes such as TA763_B (from TA763, identified in Taiwan of China, Thailand, Vietnam) and Kato_B (from Kato, identified in Japan, Taiwan of China, Thailand) were not found on the island. Third-genereation genotype JG_A (from Gilliam, identified in South Korea, Japan, Shanxi of China) and the fourth-generation Gilliam genotype (found in Inner Mongolia of China, Taiwan of China, Myanmar, Cambodia) was also not identified on Hainan Island. The detected genotypes show a broader distribution, while the undetected ones are more concentrated in specific regions like Japan, South Korea, and Taiwan of China, with some genotypes not yet widely spread in certain Southeast Asian areas.

**Fig 3:**
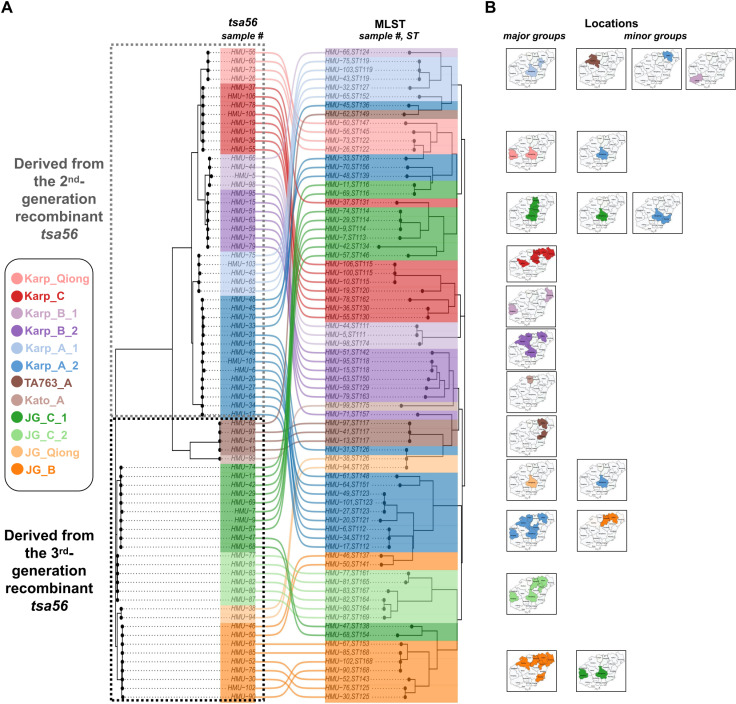
Comparative and spatial analysis of genetic data. (A) Tanglegram comparing *tsa56* hypervariable regions with middle regions of conserved genes for MLST. (B) Map displaying locations of branches/subspecies derived from phylogenetic analysis.

A tanglegram presented in Fig 3A compares the *tsa56* hypervariable regions with the middle regions of seven conserved genes used for MLST, further dividing genotypes JB_C, Karp_A, and Karp_B into two subgenotypes each (_1 and _2), resulting in a total of 12 major colored sub-branches, each representing distinct evolutionary lineages. Major sub-branches are defined by having more samples compared to their corresponding minor sub-branches with the same *tsa56* genotype, or by having no corresponding minor sub-branches at all. Ten minor sub-branches, each containing one or two samples, are observed. Interestingly, in clone group 122 (CG122), the classification of subgenotype JG_Qiong as a major sub-branch and subgenotype Karp_A_2 as a minor sub-branch suggests possible horizontal gene transfer of the *tsa56* gene, which may result in an antigenic phenotypic switch.

### Spatial diversity patterns and alpha diversity index of *O. tsutsugamushi* in the detected administrative regions

The sub-branches are mapped to specific geographic locations, including four samples with unknown resident locations and 70 samples for which information on administrative areas is available. Fig 3B presents a spatial analysis of the genetic data, with administrative areas color-coded corresponding with the sub-branches. Most minor sub-branches (8/10) are confined to either a single administrative region or neighboring administrative regions, with the exceptions being two cases in JG_C_1 and one case in Karp_C, for which location information is unavailable. The two cases in JG_C_1 (HMU-47 and HMU-68) are located in Qiongzhong and Dongfang, respectively. The variation in MLST regions may suggest the presence of two distinct lineages. Major sub-branches are spread across 1-6 administrative regions, representing at least 12 distinct lineages. Notably, Karp_A_1 and JG_Qiong are found exclusively in Qiongzhong, while Kato_A is detected only in Lingao. The Karp_Qiong lineage is observed in Dongfang and Qiongzhong, Karp_B_1 in Dongfang and Wenchang, The TA763_A in Haikou and Qionghai. The Karp_A_2 lineage is distributed across Haikou, Qiongzhong, Tunchang, Dongfang, Lingao and Danzhou indicating a broader distribution. Additional details on other lineages are shown in Fig 3B and S1 Table.

The same sequence types of *O. tsutsugamushi* were found in multiple, geographically distant locations across Hainan Island, demonstrating the long-distance transfer of these isolates. For example, CG164 associated with the JG_C_2 genotype was detected in both Qiongzhong, an inland city, and Chengmai, a northern coastal city, separated by a distance of 23.6. Similarly, CG115 associated with the Karp_C genotype was found in both Tunchang, an inland city, and Chengmai, a northern coastal city, separated by a distance of 35.6 km. Another significant example is CG114 associated with the JG_C_1 genotype, which was identified in Qiongzhong and Tunchang, both inland cities, with a transfer distance of 60.7 km. Moreover, CG112 associated with the Karp_A_2 genotype showed a long-distance transfer of 125.83 km between Lingao and Dongfang, both of which are western coastal cities. The long-distance transfer of these sequence types across the island underscores the dynamic movement and possibly complex transmission mechanisms of *O. tsutsugamushi* within Hainan Island, as detailed in S3 Table.

**Fig 4:**
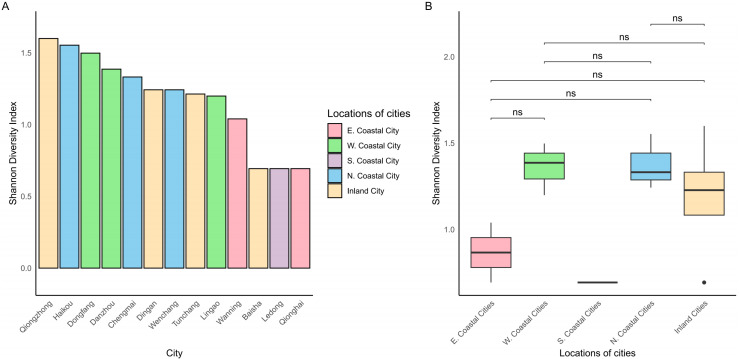
Comparison of Shannon Diversity Index across cities on Hainan Island. A. Distribution of Shannon Diversity Index; B. Regional comparison of Shannon Diversity Index.

The Shannon Diversity Index across various cities on Hainan Island shows a distinct pattern: 9 out of 14 regions, including those along the northern and western coasts as well as inland areas, exhibit higher genetic diversity, with index values ranging from 1.01 to 1.81 (Fig 4A). Notably, the inland city of Qiongzhong exhibits the highest diversity, while some southern and eastern coastal cities, such as Qionghai and Ledong, display very low or even zero diversity indices. This variation suggests that geographical location and environmental factors may influence genetic diversity. Fig 4B illustrates that northern coastal cities, western coastal cities, and inland cities have higher median diversity, with average Shannon Diversity Index values above 1.

### Variation in pathogen density dynamics across *O. tsutsugamushi* genotypes

**Fig 5:**
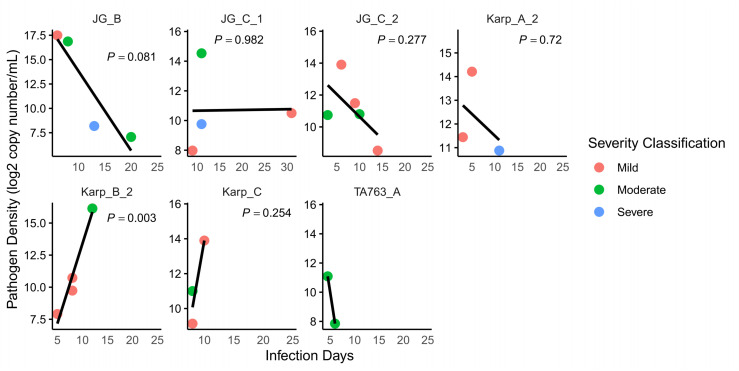
Linear regression fit of infection days on pathogen density by genotype. The linear relationship between infection days and pathogen density (log-transformed) for each genotype is illustrated. Pathogen density is quantified as the copy number of the *tsa56* gene per milliliter (mL) of blood. Each panel represents a different genotype, with scatter points indicating pathogen density in individual samples and color coding the severity of the condition (Mild, Moderate, Severe). The solid black line represents the linear regression fit for each genotype, and *P*-values for each genotype’s regression model are annotated on the plot.

In a study of 59 cases representing 12 major sub-branches, infection duration (calculated from the onset of fever to hospital admission) ranged from 3 to 31 days, with a median duration of 8 days and an average of approximately 9.7 days, based on data from 26 cases. Detailed information is available in S3 Table. This variability is illustrated in the linear regression models presented in Fig 5, which depict the relationship between fever duration and log-transformed pathogen density for each genotype. Among the analyzed genotypes, only Karp_B_2 showed a statistically significant correlation between infection duration and pathogen density (*P*-value < 0.05). In this genotype, pathogen density increased with longer infection durations, potentially indicating enhanced pathogen replication or accumulation. In contrast, JG_B and JG_C_2 showed decreasing pathogen densities over time, though these trends were not statistically significant. JG_C_1 maintained relatively stable pathogen levels throughout the infection period.

## Discussion

The genetic diversity analysis identified at least 12 distinct major genotypes of *O. tsutsugamushi* on Hainan Island. These include second-generation genotypes such as Karp_A_1, Karp_A_2, Karp_B_1, Karp_B_2, and Karp_C, as well as third-generation genotypes like JG_B, JG_C_1, JG_C_2, Kato_A, and TA763_A, all of which are prevalent across Southeast Asian countries and Taiwan of China along the East Asian-Australasian Flyway [37]. The identification of new genotypes, such as Karp_Qiong and JG_Qiong, closely related to those found in South Korea and Taiwan of China, suggests the presence of previously undescribed *tsa56* subgenotype lineages on Hainan Island.

Genetic profiling was successfully performed on 97 of 115 positive samples, while 18 samples were excluded due to insufficient or degraded DNA, likely caused by low bacterial load or suboptimal storage conditions. This highlights the need for improved sample handling in future studies. The long-distance transfer of identical sequence types between geographically distant locations, as demonstrated by the spread of ST112, ST114, ST115, and ST164 across various parts of the island, implies that *O. tsutsugamushi* may have highly dynamic movement patterns, possibly facilitated by hosts such as birds that travel significant distances. This finding raises the question of understanding the transmission mechanisms of the pathogen beyond the typical rodent hosts [44].

The Shannon Diversity Index analysis further reveals that regions with higher genetic diversity, such as Qiongzhong and other northern and western coastal cities, may act as hotspots for the emergence and spread of diverse *O. tsutsugamushi* isolates. Four of the top five regions with higher genetic diversity (Haikou, Dongfang, Danzhou, and Chengmai) contain national or provincial nature reserves, national wetland parks, or municipal/county-level nature reserves, which serve as intertidal wetlands or habitats for wintering waterbirds [45]. Interestingly, Qiongzhong exhibits the highest *O. tsutsugamushi* diversity, despite not being a key area along the migratory bird routes in 2020. Further investigation indicates that environmental changes in Qiongzhong’s Guyue Villa after 2008, a site once recognized as a critical habitat for freshwater birds like the Grey-headed Swamphen (*Porphyrio poliocephalus*) and Lesser Whistling-duck (*Dendrocygna javanica*), led to the disappearance of the Grey-headed Swamphen from Hainan island [45]. The Grey-headed Swamphen has a limited flight range, typically less than 300 km in response to environmental changes and relies on wetland habitats, such as grasslands, for feeding and living. This species was once widespread across Southeast Asia and Taiwan of China [46,47]. The Lesser Whistling-duck is also found across Southeast Asia and Taiwan of China (data available at http://www.eaaflyway.net/). The relationship between these birds and their associated mites from the family Trombiculidae is not well understood, but it could potentially play a role in the high genetic diversity of *O. tsutsugamushi* observed in Qiongzhong in the past, although further research is needed to clarify this connection.

Pathogen density analysis across different genotypes demonstrated significant variability in infection dynamics. The Karp_B_2 genotype exhibited a notable increase in pathogen density with prolonged infection duration, suggesting effecient replication during progression. In contrast, genotypes like JG_B and JG_C_2 exhibited less pronounced or decreasing trends, though additional data are needed to confirm these patterns. These findings align with previous studies showing higher *O. tsutsugamushi* DNA loads in patients infected with the Karp group compared to the Gilliam group (*P* < 0.05) [39]. Although stratified analyses for major groups like Karp and Gilliam were considered, unpublished findings from comparative genomic analysis and in vitro experiments suggest substantial genomic differences and varied replication rates among subtypes. These observations highlight the complexity of genotypic trends and suggest that broader group-level analyses may oversimplify the biological diversity within these groups. To better understand the biological relevance of these genomic and phenotypic differences, future research should integrate comprehensive clinical data and controlled analyses to disentangle genotypic effects from other contributing factors, providing deeper insights into infection dynamics.

Limitations persist regarding the representativeness of the samples, as they were collected from four hospitals in two specific regions of Hainan Island. These hospitals were chosen to represent distinct levels of economic development and ecological settings, spanning the northern provincial capital, Haikou, and the central mountainous agricultural region, Qiongzhong. While these hospitals are tertiary care facilities serving the entire province, the southern and eastern coastal regions were underrepresented in the dataset. This underrepresentation may be attributed to differences in healthcare-seeking behavior, accessibility challenges, or other regional factors affecting patient visits to these hospitals. As a result, the genetic diversity in the southern and eastern coastal regions may have been underestimated, and unique ecological or transmission dynamics in these areas were not fully captured. Furthermore, the lack of comprehensive epidemiological data limits the ability to evaluate infection trends and prevalence across the island. Despite these limitations, the findings provide valuable insights into the genetic diversity, spatial distribution, and infection dynamics of *O. tsutsugamushi* on Hainan Island. Future studies should prioritize expanding sampling efforts to include more diverse geographical regions and integrate genetic data with detailed epidemiological information. Collaborative efforts with local CDCs, particularly in underrepresented areas, will be crucial for achieving a more comprehensive understanding of transmission dynamics and for developing targeted public health interventions.

## Supporting information

S1 Table Suspected scrub typhus patients recruited in Hainan Island during June 2018 to May 2021. (XLSX)

S2 Table Summary of population averages, *Orientia tsutsugamushi* infected individuals, infection rates, diversity index, and wetland distribution in Hainan Island (2018-2020). (XLSX)

S3 Table Summary of administrative region distribution and pathogen density of *Orientia tsutsugamushi* isolates, including sequence type, clonal complex, genotype, and clinical data. (XLSX)

S1 Fig Hierarchical evolution of *tsa56* genotypes across generations and serotypes with geographical distribution. (TIF)

S2 Fig Annual infection rates based on residential locations of cases on Hainan Island (2018–2020, limited data). (TIF)
