# Supplementary figures and images for: Patient-centric analysis of Orientia tsutsugamushi spatial diversity patterns across Hainan Island, China
Source: PLoS Negl Trop Dis. 2025 Mar 18;19(3):e0012909. doi: 10.1371/journal.pntd.0012909 (PMC11918436; doi:10.1371/journal.pntd.0012909)

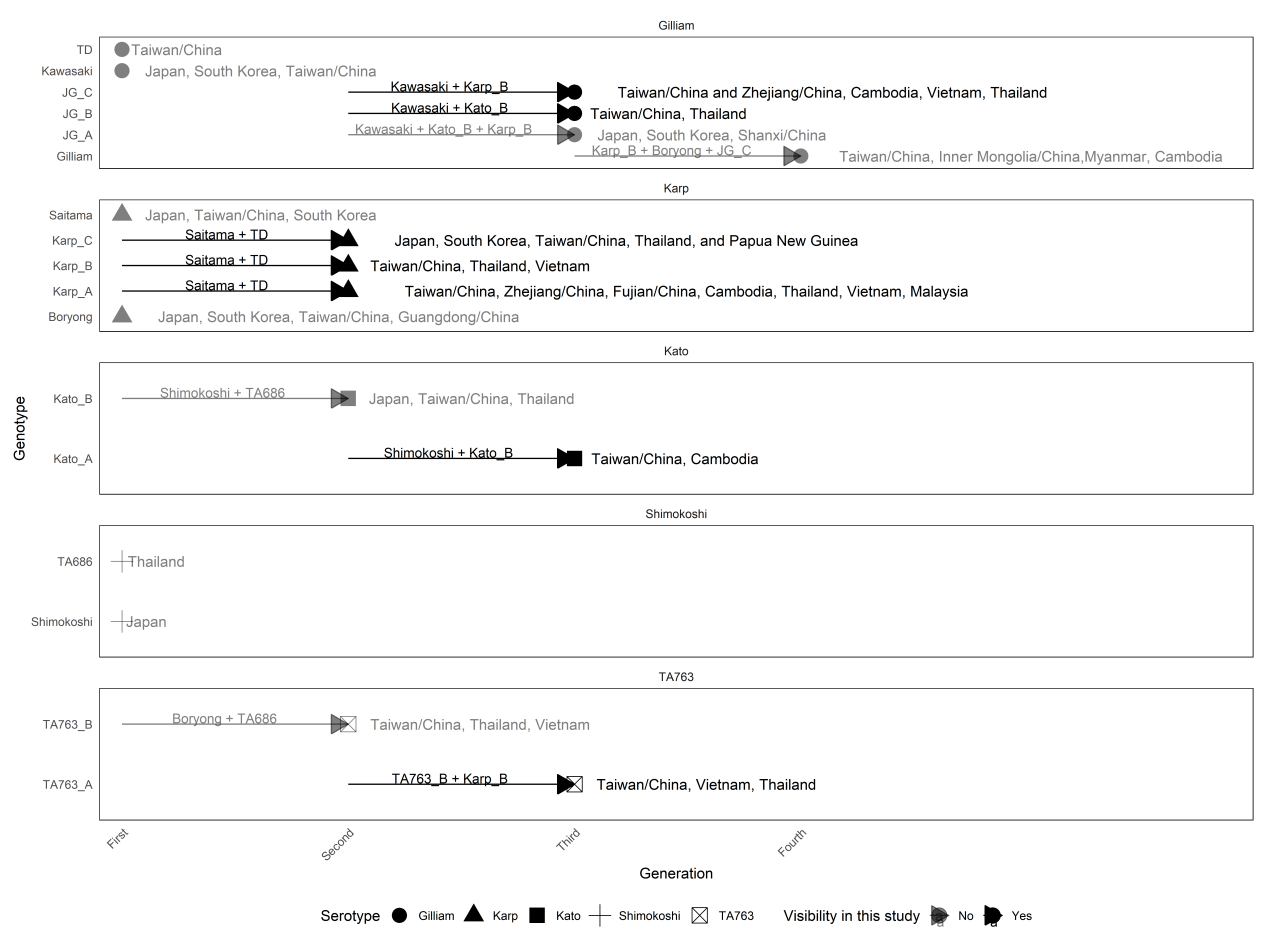

Supplement: S1 Fig — (TIF) [file pntd.0012909.s004.tif]

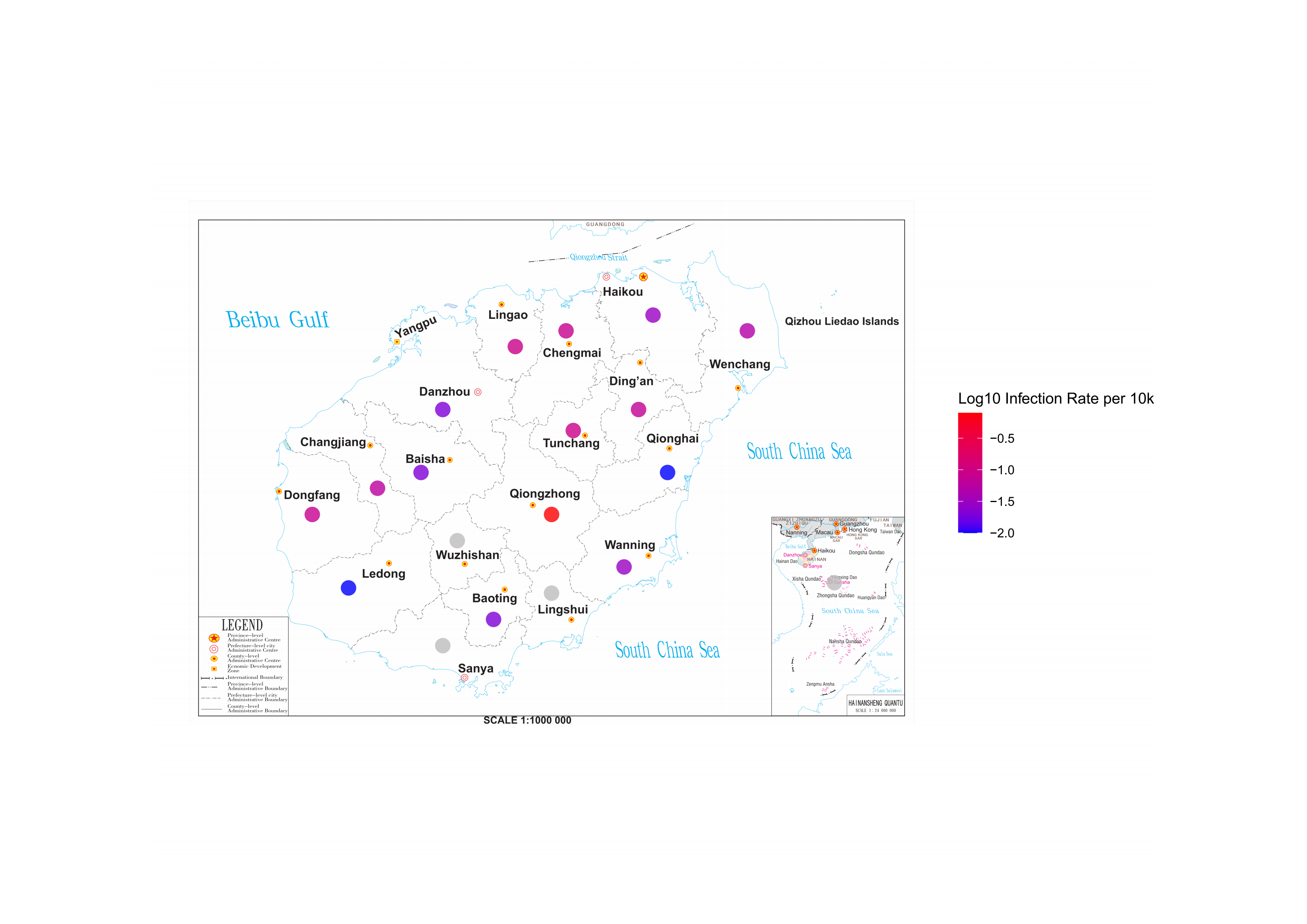

Supplement: S2 Fig — (TIF) [file pntd.0012909.s005.tif]
